# The Mechanism of Online Health Information Seeking Switching to Online Medical Consultation: Cross-Sectional Study

**DOI:** 10.2196/78397

**Published:** 2025-12-03

**Authors:** Lijiang Zhang, Jingjing Xia, Hui Chen, Yang Bai, Jun Wang, Liuan Wang, Wenjie Ren

**Affiliations:** 1 School of Health Management Henan Medical University Xinxiang, Henan China; 2 Institutes of Health Central Plains Henan Medical University Xinxiang, Henan China; 3 School of Public Administration and Policy Renmin University of China Beijing China; 4 Center for Health Policy Research and Evaluation Renmin University of China Beijing China; 5 Healthy China Institute Renmin University of China Beijing China; 6 School of Management Beijing Institute of Technology Beijing China; 7 Social Science Domain Beijing Institute of Technology Zhuhai China; 8 Qionghai Hospital of Traditional Chinese Medicine Qionghai China

**Keywords:** online health information seeking, online medical consultation, switching behavior, knowledge-attitude-practice, social support, health belief model

## Abstract

**Background:**

Internet health care plays a crucial role in addressing the challenge of distributing high-quality medical resources and promoting the optimal allocation of these resources and health equity in China. Online medical consultation (OMC) plays a more significant role than online health information seeking (OHIS). Currently, the proportion of Chinese patients using OMC is low. Therefore, it is essential to enhance patient engagement with OMC and fully leverage the role of internet health care in optimizing the allocation of medical resources.

**Objective:**

This study aims to explore the correlation mechanisms of online medical community users’ switching behaviors from OHIS to OMC.

**Methods:**

This study is based on the knowledge-attitude-practice theory, which combines the social support theory and the health belief model to construct a research model of users’ willingness to transition from OHIS to OMC. The study adopts a questionnaire survey and structural equation modeling method to conduct an empirical study.

**Results:**

Gaining knowledge about information support has a significant positive impact on perceived susceptibility (β=.339, *P<*.001), perceived severity (β=.348, *P*<.001), and perceived benefits (β=.361, *P*<.001), while having a significant negative impact on perceived barriers (β=–.285, *P*<.001). Gaining knowledge about emotional support positively affects perceived susceptibility (β=.220, *P<*.001) and perceived benefits (β=.149, *P*<.01) but does not significantly influence perceived severity (β=–.006, *P>*.05) or perceived barriers (β=.099, *P*>.05). Perceived susceptibility (β=.123, *P*<.05), perceived severity (β=.174, *P*<.001), and perceived benefits (β=.273, *P*<.001) positively influence patients’ transition to online consultation behavior, whereas perceived barriers (β=–.112, *P*<.05) negatively impact this switch. In addition, we found that gaining knowledge about information support not only directly affects patients’ behavior in switching to online consultations but also impacts patients’ OMCs through perceived susceptibility (14.23%), perceived severity (13.17%), and perceived benefits (25.28%). In contrast, gaining knowledge about emotional support does not directly influence patient behavior transfer; it operates only through perceived susceptibility (46.95%) and perceived benefit (52.90%).

**Conclusions:**

This study integrated the knowledge-attitude-practice framework, social support theory, and health belief model to uncover the internal logic of patients’ behavioral transfers within online health communities. It confirmed the mediating role of the cognitive-emotional dual-drive pathway and health beliefs. The findings provide a scientific basis for the functional design of online health care platforms and for precise health knowledge dissemination strategies.

## Introduction

### Background

The rapid integration of internet technology into the medical and health sector, along with the strategic demand for the “Healthy China” initiative, has fueled the growth of internet health care in China. Internet health care plays a crucial role in addressing the challenge of distributing high-quality medical resources and promoting the optimal allocation of these resources, as well as health equity. Among these, the online medical community is the most widely used and highly recognized model of internet health care service [1]. Patients’ behavior in using online health care communities can be categorized into free online health information seeking (OHIS) and paid online medical consultation (OMC). The former focuses on the search, acquisition, screening, and adoption of health information [2,3]. The latter emphasizes remote consultations with physicians, as well as access to treatment options [4]. Although both can enhance public health awareness and promote access to health services [5], OMC allows quality health care resources to reach grassroots and remote areas more effectively [6], generating an optimal allocation of health care resources that cannot be achieved by simply seeking health information [7]. Meanwhile, OMC plays a more significant role than OHIS in promoting health care system reform and health equity [8]. Currently, the proportion of Chinese patients using OMC is low, and the report of “the 55th Statistical Report on the Development of the Internet in China” shows that as of December 2024, the number of internet health care users in China reached 364 million, but fewer than 10% of them use internet diagnosis and treatment services. Therefore, it is essential to enhance patient engagement with OMC and fully leverage the role of internet health care in optimizing the allocation of medical resources.

The theory of switching behavior posits that users progress through a phased transformation process when engaging with online communities, typically evolving from initial information acquisition to sustained, in-depth participation, and ultimately culminating in proactive engagement behaviors [9]. In online medical communities, patients initially gather health information to assist in their health management either passively (eg, through serendipitous encounters and browsing) or actively (via intentional search and retrieval) [10,11]. However, health information on the internet originates from a variety of sources with differing reliability, which impacts patients’ evaluation and screening of health information [12]. Consequently, OHIS does not fully meet patients’ health needs, making it more difficult to obtain information with high clinical guidance value [9]. OMC is a type of medical service behavior that depends on the online medical community to facilitate disease diagnosis and treatment plan determination between the doctor and the patient, using either asynchronous or synchronous methods [13]. By using OMC services, doctors can provide patients with accurate disease diagnoses and personalized treatment plans, effectively addressing the inherent drawbacks of OHIS while more efficiently meeting individuals’ needs for high-quality online medical services. Consequently, OMC is steadily gaining popularity among patients.

However, OMC presents a challenging decision-making process. First, online medical communities gather a large number of doctors with diverse professional skills, clinical experiences, and qualification backgrounds, and patients often face significant decision-making pressure and cognitive load during the selection process. Second, health care services are typically trust commodities [14] that need to establish sufficient reliability to be selected. Compared with online decision-making behavior in commercial environments, such as online shopping, patients tend to be more cautious when choosing OMC services [15]. OHIS is an important antecedent behavior for patients taking OMC. It has been shown that if an online health care community provides a large number of useful health information resources, patients’ knowledge and sense of empowerment increase [16], and patients are more likely to choose OMC services [17]. However, there is a lack of current research on the association between the 2 behaviors, and our literature survey reveals that existing relevant research primarily focuses on 2 aspects. One is the relationship between OHIS and offline health service utilization. Some studies suggest that OHIS promotes health service utilization, while others argue that OHIS behaviors may hinder health service access utilization [18]. The second aspect is the switching behavior in online communities, and the research primarily relies on the theory of “push-pull-anchoring” to examine the formation of transfer willingness, influencing factors, and customer loyalty, among others. The study explores the transition from OHIS to OMC behavior, which is akin to the migration between products with different characteristics within the same medium [19], similar to the shift between free and paid services or the service upgrades of online platform users [20,21]. However, the first type of research overlooks the relationship between OHIS and the use of online health services such as OMC. Moreover, the mechanistic explanation proposed by some studies—that OHIS triggers health anxiety and motivates patients to seek offline consultation—does not effectively explain the relationship between OHIS and OMC. This is because health anxiety, combined with choice overload in the online health care community [22], makes it challenging to encourage OMC behavior. The second type of research primarily focuses on explaining the behavioral transfer mechanism in nonhealth domains. However, the process of health-related behavioral transfer is more complex, usually involving the precognitive belief change process and the final behavioral change process [23]. Therefore, the findings and theories of online behavioral transfer in nonhealth domains cannot adequately explain the OHIS to the OMC behavioral shift mechanism.

To address the gaps in these researches, this study notes that from a positive driver perspective, health information provided by health care professionals is perceived as authoritative and supportive [24]. In addition, online doctor-patient interactions posted on websites allow patients to perceive emotional support. Moreover, the knowledge related to this health information and interactions can increase patients’ involvement and engagement [25]. Meanwhile, the classic theory of health behavior change, knowledge-attitude-practice (KAP), can thoroughly explain the transformation of people’s cognition, beliefs, and actions. Therefore, this study proposes constructing a research framework based on the theoretical model of KAP. In addition, grounded in the theory of social support, it will serve as the foundation for the study’s theory, incorporating knowledge variables (K), individual subjective belief variables (A) rooted in the health belief model (HBM), and the transition from OHIS to OMC as behavioral variables (P). This study explores whether receiving social support and external knowledge motivates patients to take action online, and whether this relationship is mediated by their health beliefs. This core question aims to offer both theoretical and practical guidance for helping patients engage in OMCs, along with recommendations for the platform’s operation and development.

### Theoretical Basis

#### KAP Model

Originating in the field of health behavior research in the mid-20th century, KAP theory is a classic model used to explain how personal knowledge and beliefs influence behavior change. It divides human behavior change into 3 successive processes: acquiring knowledge, generating beliefs, and forming behaviors, which progress layer by layer. Knowledge serves as the foundation for behavior change, while belief acts as the driving force behind behavior change. This theory has been widely used in public health [26], health behavior, health education, and other research fields. KAP theory is suitable for depicting the behavioral change process from OHIS to OMC. When first entering an online medical community, patients often browse disease-related papers and patient comments passively due to limited trust in this community. As individuals gradually gain emotional support, accumulate knowledge, or receive practical help from other patients and health care professionals through these channels (K), they build trust in the effectiveness and timeliness of the online community’s medical consultations and diagnoses (A). This trust prompts them to use the OMC service (P). Therefore, this study is grounded in the KAP framework to explore the internal mechanisms of the health belief shift that occurs as patients access health information in the online medical community and how this leads to further online consultation behavior.

#### Social Support

Social support theory serves as the theoretical foundation for the K (knowledge) part of the KAP framework in this study. Social support refers to people’s behaviors such as emotional communication, resource sharing, and information exchange within social relationship networks [27]. This includes information support, emotional support, and instrumental support [28]. In health care research, scholars typically focus more on emotional support (40%) and informational support (31.7%) frequently [29]. Information support encompasses various types of information that individuals receive from others or social networks, aiding them in solving problems, making decisions, and understanding their surroundings. This information can include knowledge about specific matters, general experience, or advice and guidance. Emotional support refers to the psychological comfort and assistance provided to individuals through the expression of care, understanding, sympathy, encouragement, trust, and other emotional factors. This support aims to meet their emotional needs, helping them cope with stress and alleviate negative emotions [27]. The online medical community builds a social relationship network. Patients, on one hand, learn practical knowledge about disease diagnosis and treatment through health care popularization and professional question and answer. On the other hand, they communicate with other patients in the community to exchange their conditions, share experiences, and obtain emotional support. The acquisition of this experiential knowledge significantly enhances patients’ trust in the online community [30], which helps drive the adoption and use of upgraded health care services on the platform by patients.

#### Health Belief Model

HBM is a theoretical framework that explains and predicts individual health behaviors, emphasizing the essential role of cognitive and psychological factors in the decision-making process related to health behaviors. HBM argues that an individual’s adoption of a specific health behavior depends on their perceptions and beliefs about health issues. This includes factors such as perceived susceptibility, perceived severity, perceived benefits, perceived barriers, cues to action, and self-efficacy [31]. In simple terms, individuals are more likely to adopt a specific health behavior if they believe they are at risk for a disease, that the disease can lead to severe consequences, and that adopting this health behavior is effective in preventing or reducing the threat of the disease. Furthermore, they should believe they can perform the behavior. In this study, the HBM was used to illustrate the A (belief) component of the KAP model. Given that the cues to action in the HBM typically refer to external triggering factors, this study focuses on an integrated model of individual internal cognition and social support, which is less closely linked to the core of the research framework. Self-efficacy refers to confidence in one’s ability to perform a behavior. The actual online consultation platforms are continuously optimizing their interfaces, and the technical threshold is relatively low, which may not be a core influencing factor or can be indirectly reflected through perceived barriers. Therefore, in the HBM, this study includes only 4 constructs: perceived susceptibility, perceived severity, perceived benefits, and perceived barriers. Perceived susceptibility refers to an individual’s subjective assessment of the likelihood of experiencing health issues. Perceived severity is an individual’s subjective evaluation of the potential consequences of a health problem, including damage to physical functioning, impact on social functioning, and the economic burden. Perceived benefit is an individual’s belief that adopting specific health behaviors will reduce the risk of disease or lessen the consequences of disease. Perceived barriers represent individuals’ perceptions of the challenges and obstacles they face in adopting health behaviors.

### Research Model and Hypotheses

#### Supportive Information Seeking and Health Beliefs

Information support in social support theory suggests that individuals who receive information and incorporate it into their existing knowledge systems can influence the formation and transformation of related concepts and beliefs. In an online health community (OHC), patients’ access to information about doctors who provide health advice or treatment plans enhances their trust in the community. The more frequent and comprehensive their exposure to such supportive knowledge, the higher their perceived susceptibility [32]. Therefore, this study hypothesized that patients’ access to information support positively influences their perceived susceptibility (H1a). Having more health knowledge improves people’s perception of health risks and allows for a more accurate assessment of disease severity. Tang et al [21] found that individuals with higher levels of access to social support are more likely to view the consequences of a disease as more serious. Therefore, this paper argues that patients’ access to knowledge about information support positively influences their perceived severity (H1b). Moreover, it has been confirmed that seeking information online for support not only conveys knowledge about the disease but also helps patients recognize its benefits [31] and reduces perceived barriers [33], thereby positively influencing online health behaviors. Based on this, the following hypotheses are proposed: patients’ acquisition of knowledge about information support positively affects their perceived benefits and negatively impacts their perceived barriers (H1c and H1d).

Emotional support in social support theory suggests that emotional support providers may convey disease-related risk or protective messages. For example, patients in online health care communities often engage in disease risk exchanges, prompting those with access to relevant information to reflect on the likelihood of their illness. Risky narratives, such as “I ignored a symptom in the first place,” may also enhance individuals’ perceived risk susceptibility [33]. Therefore, this paper proposes the hypothesis that “patients’ acquisition of knowledge about emotional support positively affects patients’ perceived susceptibility” (H2a). Second, information based on emotional support may trigger in-depth discussions about the consequences of the disease. Patients sharing their experiences with complications in a support group may lead others to take the disease more seriously [34], affecting perceived severity. This study hypothesized that patients’ acquisition of knowledge about emotional support positively affects their perceived severity (H2b). Meanwhile, emotional support enhances recognition of the value of health behaviors, and patients are more convinced of the benefits of adopting online health care services after learning about treatment successes through the online health care community. In other words, patients’ access to knowledge about emotional support affects their perceived benefits (H2c). Finally, emotional support can help reduce perceived barriers by providing resources such as accompanying patients to medical appointments or recommending an online doctor. It also lessens the patient’s fear and anxiety about the disease indirectly [35]. Based on this, this study hypothesized that patients’ acquisition of knowledge about emotional support negatively affects their perceived barriers (H2d).

#### Health Beliefs and the OHIS-OMC Switching Behavior

**Figure 1:**
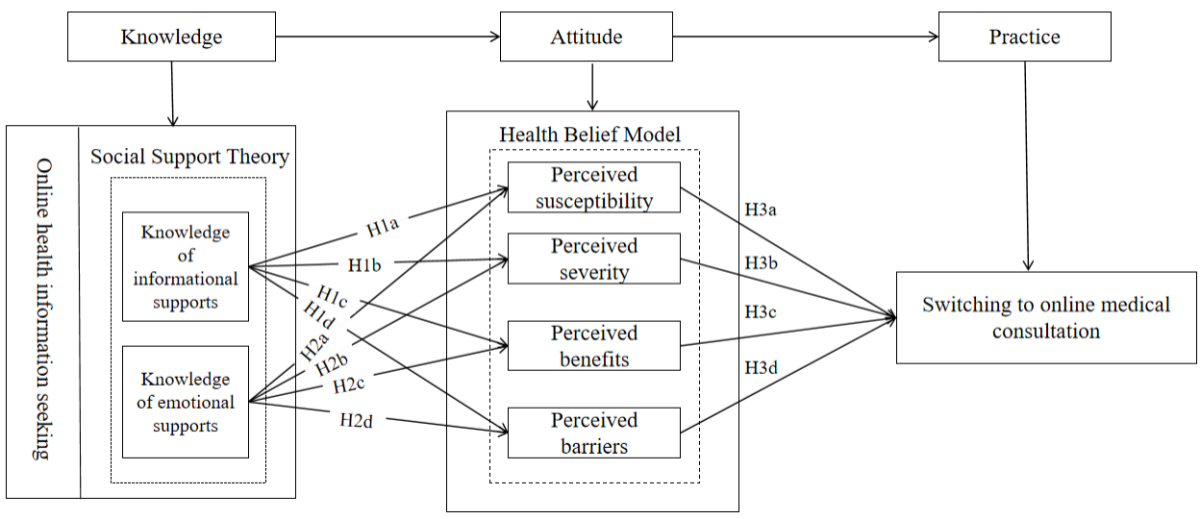
The research model.

HBM emphasizes that an individual’s subjective judgment of their likelihood of developing a disease (ie, perceived susceptibility) influences their health behavior decisions. When patients believe that they are susceptible to a disease, their worry and uncertainty prompt them to seek medical information and help [36], and their subjective judgment of the potential consequences of the disease (perceived severity)—specifically, the perceived health damage caused by the disease—significantly affects their health behaviors. Simultaneously, individuals’ assessment of the benefits they can gain from adopting a health behavior (perceived benefits) also impacts the frequency of their actions [37]. Perceived barriers represent the various obstacles that individuals anticipate when adopting a health behavior; these negative perceptions can inhibit online inquiry behavior [38]. Therefore, based on these researches, this study concludes that patients’ perceived susceptibility positively influences their transition from OHIS to OMC (H3a). In addition, patients’ perceived severity positively affects their transition from online information acquisition to online consultation (H3b). Moreover, patients’ perceived benefits positively impact their shift from OHIS to OMC (H3c), while patients’ perceived barriers negatively affect their transition from OHIS to OMC (H3d). Based on these analyses, the theoretical research model developed in this study is illustrated in Figure 1.

## Methods

### Study Design

Structural equation modeling (SEM) is a model that analyzes the path relationships among intricate variables, based on factor analysis and linear regression methods. SEM can be used to explore relationships between variables. It incorporates causal relationships among variables and measurement errors into a unified analytical framework, allowing for the simultaneous evaluation of relationships among multiple variables. These variables can be observed data (such as indicators from survey questionnaires) or latent variables (such as cognition, emotion, beliefs, and other factors that are difficult to observe directly). SEM operates at the scale level, meaning it reduces the dimensionality of scales into 1 principal component through factor analysis (transforming multiple variables into a single variable) before conducting path analysis.

### Measurement Instrument

Most of the construct items in this study were adapted from validated existing scales. Each item was measured using a 5-point Likert scale, ranging from 1=“completely disagree” to 5=“completely agree.” To ensure the scientific and content validity of the questionnaire, the measurement items were based on established scales from domestic and international studies, and some were revised to suit the online health care application scenario.

The specific measurement items and theoretical sources are shown in Table 1. Among them, seeking informational support and emotional support refer to the scales of Zhu et al [39] and Liang et al [40]. Perceived susceptibility and perceived severity refer to the scales of Noroozi et al [41]. Perceived benefits refer to the scales of Gong et al [42]. Perceived barriers refer to the scales of Huang et al [43] and Yuan and Tang [44]. OMC behavior refers to the scales of Fornell and Larcker [45].

To ensure the scientific accuracy of the questionnaire, we conducted a pilot survey. Fifty individuals who have sought online health information were invited to participate in the pilot survey. We gathered their feedback and suggestions during the completion of the initial questionnaire to further refine the instrument, resulting in the final version of the questionnaire.

**Table 1:** Constructs and measures.

Constructs and measures					References
**OHIS^a^ behavior**					[39,40]
	KIS^b^				
		KIS1: While browsing the online health community, I discovered that the platform’s doctors assist patients in identifying the causes of their diseases.			
		KIS2: While browsing the online health community, I discovered that the platform’s doctors provided effective responses and suggestions to patients.			
		KIS3: While browsing the online health community, I discovered that the platform’s doctors would provide problem-solving information to patients.			
		KIS4: While browsing the online health community, I found that the doctors on the platform would provide specific solutions to patients.			
	KES^c^				
		KES1: While browsing the online health community, I felt the doctor’s concern for the patient.			
		KES2: While browsing the online health community, I feel that doctors comfort and encourage their patients.			
		KES3: While browsing the online health community, I felt that the doctor would confront health problems alongside the patient.			
		KES4: While browsing the online health community, I felt that the doctor was attentive to the patient’s feelings.			
**Health belief model**					[41-44]
	PSU^d^				
		PSU1: In everyday life, people frequently face health issues.			
		PSU2: When I maintain an unhealthy lifestyle for an extended period, I feel my chances of getting sick increase.			
		PSU3: The chances of developing a disease increase when I am unwell.			
		PSU4: I may become ill if I don’t seek medical advice promptly for concerning symptoms in my body.			
	PSE^e^				
		PSE1: I feel more anxious and afraid when I consider the possibility of developing a severe disease.			
		PSE2: My daily life would be impacted if I developed a serious illness.			
		PSE3: If I don't seek medical advice quickly, I'm worried that the disease may get worse.			
	PBE^f^				
		PBE1: Online consultations are beneficial to my health.			
		PBE2: Online consultations are beneficial for my health management.			
		PBE3: Online consultations will help me detect illnesses in advance.			
		PBE4: Online consultation will help alleviate my worries about my condition.			
	PBA^g^				
		PBA1: I am concerned that the private health information submitted to online doctors will be misused.			
		PBA2: I worry that the quality of the online consultation service is not guaranteed.			
		PBA3: I am concerned about excessive use of online consultation services.			
		PBA4: I am concerned about my ability to accurately communicate my consultation needs to community doctors.			
**From OHIS to OMC**					[43]
	OMC^h^				
		OMC1: I will consider consulting an online medical community physician if I experience health issues in the future.			
		OMC2: In the future, when I encounter health problems, I will consult with online medical community doctors as much as possible.			
		OMC3: In the future, when I face health issues, I plan to consult a doctor from an online medical community.			

^a^OHIS: online health information seeking.

^b^KIS: knowledge of informational support.

^c^KES: knowledge of emotional support.

^d^PSU: perceived susceptibility.

^e^PSE: perceived severity.

^f^PBE: perceived benefits.

^g^PBA: perceived barriers.

^h^OMC: online medical consultation.

### Ethics Considerations

This study was approved by the ethics committee of Xinxiang Medical University (20250478). Prior to the survey, each participant completed an informed consent form and agreed to the analysis of the obtained data by the research team. The data collection and storage processes in this study strictly adhered to confidentiality principles, ensuring the anonymization of personal information. This study did not involve the use of images or photographs of the participants. Participants were eligible to receive compensation ranging from US $1 to US $2 for their involvement in the study.

### Data Collection

Our survey targets the population that has used the internet to search for health information. The target population of this study includes Chinese adults with experience or familiarity with digital health technologies. The eligibility criteria require participants to be aged at least 19 years and confirm at least one of the following: (1) previous use of health-related technologies (such as health apps or smart wearables), (2) functional understanding of the concept of digital health, or (3) indication of willingness to use technology for personal or health management. These criteria ensure that respondents possess the necessary background knowledge to provide meaningful and effective responses to the survey items. We created the questionnaire using the software “Questionnaire Star” [Changsha Ranxing Information Technology Co, Ltd] and distributed it through 2 primary channels. After the preresearch was approved, we officially distributed our questionnaires. The recruited participants would receive a random cash red envelope, with amounts ranging from US $1 to US $2. First, we shared the questionnaire within Chinese communities, such as “QQ groups” and “Douban groups” for users of OHCs. We also recruited participants through OHCs such as “Baidu Tieba” and “Haodaifu Online.” A total of 509 questionnaires were collected from March to May 2024. After excluding incomplete and invalid responses according to the eligibility criteria, we obtained 425 valid questionnaires.

### Statistical Analysis

In this study, we used SEM to analyze the collected data and evaluate the research model. Following a 2-step procedure, we first used SPSS (version 26.0; IBM Corp) software to examine the measurement model, ensuring its reliability and validity. Tables 2 and 3 report the reliability and validity results. As Table 2 shows, the Cronbach α and composite reliability values were greater than the proposed threshold of 0.7 [45], indicating qualified reliability. Convergent validity was assessed by the item loadings and the average variance extracted (AVE) from expected constructs. The results show that AVEs were higher than the recommended value of 0.5 [46], and all indicator loadings exceeded the threshold of 0.7, suggesting satisfactory convergence validity. Table 3 suggested that the square root of AVE values for each construct exceeded all its correlation coefficients with other constructs, indicating promising discriminant validity [47].

We then used AMOS (version 26.0; IBM Corp) software to examine the fitness between the sample data model and the research model. The results are shown in Table 4, indicating that the model’s fitness indicators, including the absolute fitness indicator, value-added fitness indicator, and simple fitness indicator, all achieve a reasonable level, demonstrating that the model fits well.

**Table 2:** Validated factor analysis results.

Constructs	Cronbach α	Convergent validity AVE^a^	Combined reliability
KIS^b^	0.802	0.508	0.804
KES^c^	0.814	0.539	0.822
PSU^d^	0.832	0.568	0.839
PSE^e^	0.809	0.598	0.816
PBE^f^	0.821	0.557	0.832
PBA^g^	0.825	0.556	0.831
OMC^h^	0.813	0.617	0.825

^a^AVE: average variance extracted.

^b^KIS: knowledge of informational support.

^c^KES: knowledge of emotional support.

^d^PSU: perceived susceptibility.

^e^PSE: perceived severity.

^f^PBE: perceived benefits.

^g^PBA: perceived barriers

^h^OMC: online medical consultation.

**Table 3:** Results of distinction validity test^a^.

Constructs	OMC^b^	PBA^c^	PBE^d^	PSE^e^	PSU^f^	KES^g^	KIS^h^
OMC	0.785	N/A^i^	N/A	N/A	N/A	N/A	N/A
PBA	0.206	0.745	N/A	N/A	N/A	N/A	N/A
PBE	0.357	0.132	0.746	N/A	N/A	N/A	N/A
PSE	0.291	0.161	0.265	0.773	N/A	N/A	N/A
PSU	0.272	0.315	0.257	0.342	0.754	N/A	N/A
KES	0.181	0.209	0.288	0.123	0.342	0.734	N/A
KIS	0.316	0.307	0.403	0.311	0.387	0.508	0.713

^a^Values on the diagonal are the square root of average variance extracted, and the lower triangles are the correlation coefficients between the factors.

^b^OMC: online medical consultation.

^c^PBA: perceived barriers.

^d^PBE: perceived benefits.

^e^PSE: perceived severity.

^f^PSU: perceived susceptibility.

^g^KES: knowledge of emotional support.

^h^KIS: knowledge of informational support.

^i^N/A: not applicable.

**Table 4:** Results of the model fitness test.

Fitness indicators			Judgment criteria		Indicator value		Adaptation effect
**Absolute fitness indicator**							
	CMIN/DF^a^	<3		1.206		Ideal	
	SEA^b^	<0.08		0.022		Ideal	
	GFI^c^	>0.9		0.943		Ideal	
	AGFI^d^	>0.8		0.928		Ideal	
**Value-added fitness indicator**							
	CFI^e^	>0.9		0.987		Ideal	
	IFI^f^	>0.9		0.987		Ideal	
	TLI^g^	>0.9		0.985		Ideal	
**Simple fitness indicator**							
	PGFI^h^	>0.5		0.747		Ideal	
	PNFI^i^	>0.5		0.794		Ideal	

^a^CMIN/DF: chi-square/degrees of freedom.

^b^SEA: standardized error of approximation.

^c^GFI: Goodness-of-Fit Index.

^d^AGFI: Adjusted Goodness-of-Fit Index.

^e^CFI: Comparative Fit Index.

^f^IFI: Incremental Fit Index.

^g^TLI: Tucker-Lewis Index.

^h^PGFI: Parsimony Goodness-of-Fit Index.

^i^PNFI: Parsimony Normed Fit Index.

## Results

### Characteristics of Respondents

The characteristics of the respondents are illustrated in Table 5. The participants’ gender was relatively balanced, with 54.40% (231/425) female and 45.6% (194/425) male. The age range covered individuals from younger than 20 years to older than 50 years. Approximately half (227/425, 53.4%) of the participants held college degrees. In terms of careers, 32.2% (137/425) of the participants were students, while 66.4% (282/425) were used. The respondents’ places of residence varied significantly, with 72.7% (309/425) of participants living in urban areas and 27.3% (116/425) residing in rural areas.

**Table 5:** Characteristics of respondents.

Variable			Participants (N=425)
**Sex, n (%)**			
	Male	194 (45.60)	
	Female	231 (54.40)	
**Age (years), n (%)**			
	≤19	20 (4.70)	
	20-29	256 (60.20)	
	30-39	47 (11.10)	
	40-49	91 (21.40)	
	≥50	11 (2.60)	
**Education level, n (%)**			
	High school and below	57 (13.40)	
	Associate degree	98 (23.10)	
	Bachelor’s degree	227 (53.40)	
	Master’s degree and above	43 (10.10)	
**Employment status, n (%)**			
	Student	137 (32.20)	
	Working/freelance	282 (66.40)	
	Other	6 (1.40)	
**Place of residence, n (%)**			
	Rural	116 (27.30)	
	Urban	309 (72.70)	

### Structural Model

#### Path Coefficients

We adopted the AMOS (version 26.0) software to examine the structural model’s path coefficients and corresponding significance levels. Table 6 shows the results of the hypothesized path analysis. Among these, seeking knowledge of information support has a significant positive effect on perceived susceptibility (β=.339, *P*<.001), perceived severity (β=.348, *P*<.001), and perceived benefits (β=.361, *P*<.001), while also having a significant negative effect on perceived barriers (β=–.285, *P*<.001). Thus, hypotheses H1a, H1b, H1c, and H1d are valid. Seeking knowledge of emotional support has a significant positive effect on perceived susceptibility (β=.220, *P*<.001) and perceived benefits (β=.149, *P*<.01), confirming hypotheses H2a and H2c. However, it showed no significant effect on perceived severity (β=–.006, *P*>.05) or perceived barriers (β=.099, *P*>.05), meaning hypotheses H2b and H2d were not established. Perceived susceptibility (β=.123, *P*<.05), perceived severity (β=.174, *P*<.001), and perceived benefits (β=.273, *P*<.001) positively affect patients’ switching to online consultation behavior, while perceived barriers (β=–.112, *P*<.05) negatively affect switching behavior, validating hypotheses H3a, H3b, H3c, and H3d.

**Table 6:** Results of hypothesized path analysis.

Hypothesis	Path correlation	Path factor	Results
H1a	KIS^a^ → PSU^b^	0.339^c^	Supported
H1b	KIS → PSE^d^	0.348^c^	Supported
H1c	KIS → PBEe	0.361^c^	Supported
H1d	KIS → PBA^f^	–0.285^c^	Supported
H2a	KES^g^ → PSU	0.220^c^	Supported
H2b	KES → PSE	–0.006	Not supported
H2c	KES → PBE	0.149^h^	Supported
H2d	KES → PBA	0.099	Not supported
H3a	PSU → OMC^i^	0.123^j^	Supported
H3b	PSE → OMC	0.174^c^	Supported
H3c	PBE → OMC	0.273^c^	Supported
H3d	PBA → OMC	–0.112^i^	Supported

^a^KIS: knowledge of informational support.

^b^PSU: perceived susceptibility.

^c^*P*<.001 (very high level of significance).

^d^PSE: perceived severity.

^e^PBE: perceived benefits.

^f^PBA: perceived barriers.

^g^KES: knowledge of emotional support.

^h^*P*<.01 (high level of significance).

^i^OMC: online medical consultation.

^j^*P*<.05 (a level of significance).

#### Mediating Effect Test

This study uses model 4 in the Process macro (SPSS [version 26; IBM Corp]) and performs a mediating effect test using 5000 bootstrap resamples to examine the mediating role of health beliefs. As shown in Table 7, both the total effect (β=.339) and the direct effect (β=.135) are significant, as the confidence interval obtained from the bootstrap analysis for the direct effect does not include zero [48]. Among the indirect effects, the confidence intervals of 3 out of 4 effects do not include zero, while the confidence interval of perceived barriers does encompass zero. This indicates that information-supported knowledge acquisition can not only directly affect patients’ behavior of switching to online consultation but also influence patients’ OMC behavior through perceived susceptibility (14.23%), perceived severity (13.17%), and perceived benefits (25.28%), as these 3 play partial mediating roles in the model path.

Since the path analysis indicates that the acquisition of emotional support knowledge has no significant impact on perceived severity and perceived barriers (H2b and H2d), when conducting the mediating effect test of the HBM related to the acquisition of emotional support knowledge and behavioral transfer, only the acquisition of emotional support knowledge, perceived susceptibility, and perceived benefit are simultaneously included in the regression analysis. The test results are shown in Table 8. The upper and lower limits of the 95% confidence intervals from the Bootstrap for both the total and indirect effects of emotional support knowledge acquisition on patient behavior transfer do not include 0, whereas the upper and lower limits of the 95% confidence intervals from the Bootstrap for the direct effect do contain. This indicates that the acquisition of emotional support knowledge cannot directly influence patient behavior transfer; it can operate only through 2 mediating variables: perceived susceptibility and perceived benefit. Furthermore, perceived susceptibility and perceived benefit entirely mediate the hypothetical path, accounting for 46.95% and 52.90% of the total effect, respectively.

**Table 7:** Mediating effect of health belief on the relationship between information supports knowledge seeking and switching behavior.

			Effect values		BootLLCI		BootULCI		Relative mediated effect
Total effect			0.339		0.215		0.464		N/A^a^
Direct effect			0.135		0.004		0.266		N/A
**Indirect effect**									
	Perceived susceptibility	0.048		0.008		0.098		14.23%	
	Perceived severity	0.045		0.003		0.094		13.17%	
	Perceived benefits	0.086		0.040		0.144		25.28%	
	Perceived barriers	0.026		–0.010		0.067		N/A	

^a^N/A: not applicable.

**Table 8:** Mediating effect of the health belief between Information supports knowledge seeking and switching behavior.

		Effect values	BootLLCI	BootULCI	Relative mediated effect
Total effect		0.138	0.036	0.239	N/A^a^
Direct effect		0.000	–0.103	0.103	N/A
**Indirect effect**					
	Perceived susceptibility	0.065	0.027	0.114	46.95%
	Perceived benefits	0.073	0.036	0.120	52.90%

^a^N/A: not applicable.

## Discussion

### Principal Findings

Drawing on the KAP theoretical framework, this study combines social support theory and the HBM to examine the mechanism underlying patients’ transition from seeking online health information to engaging in OMC within an online medical community. The research found that the OHIS has a positive influence on both informational and emotional support knowledge, thereby promoting OMC. In this process, perceived susceptibility, perceived severity, and perceived benefit within the health beliefs model have a partial mediating effect, while the mediating role of perceived barriers is not significant. This finding supports the underlying research hypothesis proposed in this study, suggesting that patients’ online access to positive health information can motivate them to move from OHIS to OMC through shifts in health beliefs.

First, this finding suggests that patients are inclined to acquire positive information and develop health-related knowledge when conducting OHIS. This aligns with a study by Sassenberg and Greving [49]. In their research, a survey of chronic patients indicated that individuals were more likely to obtain positive and supportive information and form favorable views about their health when using the internet for information seeking compared with other information sources.

Second, our study found that patients can positively influence perceived susceptibility and perceived benefit in health beliefs by accessing informational support and emotional knowledge. Both factors play a mediating role in facilitating patients’ transition to OMC behavior. On one hand, this suggests that patients can obtain supportive health information through online searches, which helps them observe and evaluate their own health status, thereby increasing their perception of illness [50]; on the other hand, accessing emotionally supportive information heightens their concern for their health status and significantly enhances their perceived susceptibility to disease. Perceived susceptibility, a core variable in health behavioral decision-making, directly affects individuals’ demand for specialized medical services. This explains why patients are prompted to seek further specialized online consultations, aligning with the study Li et al [51] on “Perceived susceptibility enhances the use of internet hospitals.” Meanwhile, regarding perceived benefits, accessing supportive health information enables patients to better understand, evaluate, and apply professional medical insights. In addition, emotional support strengthens the close relationships among patients and reinforces their recognition of online doctors’ professionalism and trust in service quality, all of which enhance patients’ perceived benefits. Increased perceived gains foster doctor-patient trust [52], providing a driving force for patients to adopt OMC behaviors. Furthermore, the results did not support H2b and H2d, indicating that the acquisition of emotional support knowledge had no significant effect on perceived severity or perceived barriers. This may be because emotional support primarily offers psychological comfort and empathy rather than concrete cognitive information about disease severity. The emotional exchange provides patients with more emotional relief and psychological support rather than an objective assessment of potential health consequences. On the other hand, perceived barriers are often related to practical factors such as technical difficulties, privacy concerns, and cost issues. Although emotional support can alleviate anxiety, it is less likely to directly alter patients’ perceptions of these practical obstacles. Therefore, while emotional support can enhance perceived susceptibility and perceived benefits, its impact on perceived severity and perceived barriers remains limited.

Meanwhile, this study revealed the differential mediating effect of perceived severity in the HBM. Specifically, patients’ access to systematic, information-supportive knowledge of causes, symptoms, and treatment options [53] can enhance their perception and judgment of disease severity. Perceived severity mediates the relationship between patients’ access to information-supportive knowledge and their shift to OMC behavior, which is theoretically consistent with the mechanism of facilitating online counseling use in internet hospitals, as noted in a previous study [51]. However, the results of this study also suggest that while knowledge of emotional support can generate positive health beliefs, it does not influence perceived severity, nor change perceived barriers. This contrasts with some literature that suggests that “emotional support may weaken perceived health risks” [54].

Finally, our study also found that while access to information supporting knowledge made it easier for patients to understand physicians’ responses and recommendations, reducing perceived barriers, these same barriers prevented patients from shifting to OMC. However, perceived barriers did not mediate the effect, possibly because access to information supporting knowledge had a more substantial influence on perceived susceptibility to severity and benefits, leading patients to prefer those options that could provide immediate diagnosis and treatment through convenient and efficient online consultation methods, counteracting the hindering effect of perceived barriers.

### Implications and Limitations

Based on our limited literature review, this study, for the first time, draws on the KAP theoretical framework, integrates the social support theory and the HBM, and adopts an empirical research methodology to explore the mechanisms associated with the transfer of patients’ behaviors from online health information acquisition to online consultation. This study also provides practical insights for online health care platforms. From the perspective of information support, online medical communities should encourage medical professionals to engage more in communication and interaction and regularly release authoritative and accurate health information and disease knowledge to enhance patients’ understanding of diseases and promote their in-depth utilization of online medical platform functions. From the perspective of emotional support, online medical communities should encourage patients to share their treatment experiences and psychological feelings with one another and also strengthen the emotional bond between doctors and patients. Moreover, they should develop incentive mechanisms to motivate patients to take the initiative in communicating with their doctors, such as launching point reward activities and tutorials for novice medical practitioners, to mitigate the wait-and-see approach of patients in OMCs [55]. Based on the finding that “informational support significantly influences perceived severity, while emotional support does not,” we recommend that platforms emphasize different types of content at various stages of facilitating consultation conversion: during the user’s disease awareness phase, push authoritative and systematic disease knowledge (informational support); during the user’s decision-hesitation phase, showcase warm doctor-patient interaction stories (emotional support) to enhance trust and perceived benefits. In addition, the online medical community should enhance the performance reward and punishment mechanism for doctors, encourage medical professionals to proactively answer patients’ inquiries, and demonstrate a profound commitment to humanistic care. This not only establishes trust between doctors and patients but also significantly improves patients’ continued adoption and deeper application of online medical services.

This study also has certain limitations, primarily reflected in 3 aspects that can serve as a reference for future research. First, the research data in this paper come from questionnaire surveys, and the subjectivity of users in completing the questionnaires may introduce bias into the research results. The sample primarily consists of young, middle-aged, and urban users, which may overlook the behavioral differences of older or rural groups due to the digital divide. In addition, this study predominantly sampled individuals with at least an undergraduate education; expanding the scope of survey participants in future studies would be beneficial. Finally, this study focused on the transition from acquiring health information to engaging in consultation behavior. Future research could further explore the mechanisms influencing online consultation behavior from alternative perspectives or through mixed methods. Furthermore, the use of cross-sectional data makes it challenging to capture the dynamic process of behavioral change, and the mechanisms could be better understood by incorporating longitudinal designs or mixed methods in future research.

### Conclusions

This study integrated the knowledge-attitude-behavior framework, social support theory, and HBM to uncover the internal logic of patients’ behavioral transfer within OHCs. It confirmed the mediating role of the cognitive-emotional dual-drive pathway and health beliefs. The findings provide a scientific basis for the functional design of online health care platforms and the precise formulation of health knowledge dissemination strategies. In addition, they highlight the need for future attention to the differences in digital literacy among various groups to promote the deeper development and widespread application of digital health services.

## Abbreviations

AVE: average variance extracted
HBM: health belief model
KAP: knowledge-attitude-practice
OHC: online health community
OHIS: online health information seeking
OMC: online medical consultation
SEM: structural equation modeling

## Data Availability

The datasets generated and analyzed during this study are available from the corresponding author on reasonable request.
