# Correction: The Mechanism of Online Health Information Seeking Switching to Online Medical Consultation: Cross-Sectional Study

**DOI:** 10.2196/94748

**Published:** 2026-03-16

**Authors:** Lijiang Zhang, Jingjing Xia, Hui Chen, Yang Bai, Jun Wang, Liuan Wang, Wenjie Ren

**Affiliations:** 1 School of Health Management Henan Medical University Xinxiang, Henan China; 2 Institutes of Health Central Plains Henan Medical University Xinxiang, Henan China; 3 School of Public Administration and Policy Renmin University of China Beijing China; 4 Center for Health Policy Research and Evaluation Renmin University of China Beijing China; 5 Healthy China Institute Renmin University of China Beijing China; 6 School of Management Beijing Institute of Technology Beijing China; 7 Social Science Domain Beijing Institute of Technology Zhuhai China; 8 Qionghai Hospital of Traditional Chinese Medicine Qionghai China

In “The Mechanism of Online Health Information Seeking Switching to Online Medical Consultation: Cross-Sectional Study” [1], the authors noted one error.

The equal contribution designation has been added to the following authors:

Yang Bai, Wenjie Ren

The correction will appear in the online version of the paper on the JMIR Publications website, together with the publication of this correction notice. Because this was made after submission to PubMed, PubMed Central, and other full-text repositories, the corrected article has also been resubmitted to those repositories.
